# Spatial Attention-Based 3D Graph Convolutional Neural Network for Sign Language Recognition

**DOI:** 10.3390/s22124558

**Published:** 2022-06-16

**Authors:** Muneer Al-Hammadi, Mohamed A. Bencherif, Mansour Alsulaiman, Ghulam Muhammad, Mohamed Amine Mekhtiche, Wadood Abdul, Yousef A. Alohali, Tareq S. Alrayes, Hassan Mathkour, Mohammed Faisal, Mohammed Algabri, Hamdi Altaheri, Taha Alfakih, Hamid Ghaleb

**Affiliations:** 1 Centre of Smart Robotics Research (CS2R), King Saud University, Riyadh 11543, Saudi Arabia; muneer.al-hammadi@ntnu.no (M.A.-H.); mmalsulaiman@ksu.edu.sa (M.A.); mmekhtiche@ksu.edu.sa (M.A.M.); aabdulwaheed@ksu.edu.sa (W.A.); yousef@ksu.edu.sa (Y.A.A.); mathkour@ksu.edu.sa (H.M.); m.naji@kcst.edu.kw (M.F.); malgabri@ksu.edu.sa (M.A.); haltaheri@ksu.edu.sa (H.A.); talfakih@ksu.edu.sa (T.A.); hghaleb@ksu.edu.sa (H.G.); 2 Department of Civil and Environmental Engineering, Faculty of Engineering, Norwegian University of Science and Technology, Høgskoleringen 1, 7034 Trondheim, Norway; 3 Computer Engineering Department, College of Computer and Information Sciences, King Saud University, Riyadh 11543, Saudi Arabia; 4 Computer Science Department, College of Computer and Information Sciences, King Saud University, Riyadh 11543, Saudi Arabia; 5 Department of Special Education, College of Education, King Saud University, Riyadh 11543, Saudi Arabia; talrayes@ksu.edu.sa; 6 Center of AI & Robotics, Kuwait College of Science and Technology (KCST), Kuwait City 35004, Kuwait; 7 Software Engineering Department, College of Computer and Information Sciences, King Saud University, Riyadh 11543, Saudi Arabia

**Keywords:** sign language recognition, graph convolutional neural network (GCN), attention, deep learning

## Abstract

Sign language is the main channel for hearing-impaired people to communicate with others. It is a visual language that conveys highly structured components of manual and non-manual parameters such that it needs a lot of effort to master by hearing people. Sign language recognition aims to facilitate this mastering difficulty and bridge the communication gap between hearing-impaired people and others. This study presents an efficient architecture for sign language recognition based on a convolutional graph neural network (GCN). The presented architecture consists of a few separable 3DGCN layers, which are enhanced by a spatial attention mechanism. The limited number of layers in the proposed architecture enables it to avoid the common over-smoothing problem in deep graph neural networks. Furthermore, the attention mechanism enhances the spatial context representation of the gestures. The proposed architecture is evaluated on different datasets and shows outstanding results.

## 1. Introduction

Sign language is the main communication form in deaf communities. It conveys the complex semantics of language through highly structured manual and non-manual components. The manual component is encoded in the configuration of hand fingers and the relative movements of the hands to the signer body. The non-manual components on the other hand are encoded in the micro-motions of the facial expressions and head postures [1].

There are two types of sign language gestures: static and dynamic. Static gestures mainly refer to the fingerspelling of an individual alphabet and numerical characters, which involve a fixed fingers’ configuration and fixed hand location. Dynamic gestures on the other hand might be isolated words or continuous sentences [2]. This work addresses the problem of isolated hand gesture recognition in RGB videos.

The sign language recognition systems follow one of two approaches: contact-based systems and contactless-based systems. In the contact-based systems, the gesture data are collected as a signal that represents the body movements or a sequence of body poses represented by the skeletal joints’ locations [3,4]. The classification models of such sparse data could be lightweight architectures. Consequently, the computation cost is suitable for tiny devices, which may increase the potential of their daily use. However, the performance of conventional machine learning tools on such data is low.

Another main drawback for the contact-based systems is that the user should be accustomed to some hardware setup to collect the gestures, which makes it inconvenient and uncomfortable. This restriction is avoided in contactless-based systems. The most common form of systems in this category is the vision-based systems, which utilize imaging devices such as RGB and RGB-D cameras to record the gestures [5].

The current advancement in deep learning techniques achieves promising performance not only in vision-based sign language recognition but also in many other vision-based fields such as object detection, image classification, and action recognition [6,7]. 

It is also applied in other fields such as human–robot interaction and smart healthcare systems. For instance, an incremental learning based on deep CNN is applied for human-like kinematic mapping on the robot and achieved interesting online prediction [8]. On the other hand, a multimodal ECG pattern monitoring system is proposed as a possible cybertwin-based network for the next generation of cellular networks [9]. However, the performance of conventional deep learning techniques on sign language recognition is still less than their performance in other computer vision applications. This incapability in the case of sign language recognition is attributed to two reasons.

- Scarcity of large, labeled sign language datasets.
- The representation learning of signs by conventional CNN is inefficient. It involves a lot of non-relevant patterns in the scene, while the relevant patterns are only in the movement field of the signer’s hands.

A few years ago, researchers started utilizing graph neural networks for sign language recognition. They have been encouraged by the success achieved by deep architectures such as Openpose in human pose estimation [10]. These architectures can accurately estimate the human body’s skeletal joints, which are used to construct robust graph representations for sign language gestures. This representation helps in avoiding the non-relevant patterns in the scene as well as significantly reduces the need for labeled data [11]. However, the Openpose-based architectures are slow and not useful for constructing real-time recognition systems. 

To address the mentioned issues of data scarcity, the high complexity, and the inefficiency of conventional CNN representation, a shallow graph-based 3DCNN architecture with a spatial attention mechanism is proposed in this work for sign language recognition. The main contributions in this work are as follows:

1. A novel graph-based architecture of separable 3DCNN layers is proposed for sign language recognition, with the following characteristics:
  - Effective embedding for sign language gesture features with attention-based context enhancement for the spatial representation.
  - Ability to avoid the representation over smoothing via minimizing the number of messages passing between the graph nodes.
  - Optimum computation cost by reducing the number of layers as well as utilizing an efficient skeletal estimator.
2. The performance of the proposed architecture is demonstrated on different datasets with highly varied characteristics.

The proposed architecture is based on the raw RGB videos without extra preprocessing, a need for colored gloves, or any costly and complex setup for gesture data recording. It only relies on the efficient estimation of input skeletal data, which leads to a robust graph representation. This representation is not only immunized against the bad effects of noisy and cluttered background, light variation, and signer clothing style but also able to avoid all non-relevant patterns in the scene.

The rest of this paper is organized as follows. Section 2 overviews the related work in sign language recognition. Section 3 describes the dataset used for evaluation. The proposed architecture is presented in Section 4. Then, Section 5 presents experimental results and discussion. Finally, conclusions are presented in Section 6.

## 2. Related Work

The vision-based sign language recognition addresses the problem in three phases: extracting the visual patterns locally in each frame, temporal dependence modeling, and classification. The oldest works utilized various hand-crafted feature extraction techniques such as SIFT [12,13], HOG [14,15], and the frequency domain features [16]. Other techniques such as hidden Markov models (HMM) [17] and dynamic time warping (DTW) [18] were then employed for temporal dependence modeling and conventional classifiers such as support vector machine (SVM) [19] for sign prediction. These works were mainly evaluated on small and private datasets. In a recent study, Kullback–Leibler divergence HMM was also utilized for modeling the information of hand movement. It was demonstrated that there is a performance gap between language-dependent and language-independent modeling scenarios [20]. 

Like other computer vision applications, most of the recent works exploited the advancement in deep CNN architectures for learning holistic features from each frame. Recurrent neural networks, on the other hand, were utilized for modeling the temporal dependence between consecutive frames. For example, Okan et al. applied inception architecture on both RGB frames and optical flow for sign language recognition [21]. Chen et al. employed ResNet architecture for encoding the video frames’ features in a 2D matrix before classification [22]. Aly et al. utilized semantic segmentation to detect the hands and LSTM for sequence modeling. Furthermore, 3DCNN was also intensively utilized in different ways for learning the spatio-temporal features of sign language from RGB sequence or depth map [23,24,25]. 

Recently, Qin et al. presented a system of dual-channel video transformer network (VTN) with bidirectional LSTM for continuous sign language recognition [26]. The two channels of the VTN are trained on RGB video and RGB video difference of isolated gestures and then used as a feature extractor for the continuous gestures. The bidirectional LSTM, on the other hand, is used for modeling the sequence dependence. The proposed model might be enhanced by a language model to scale well for continuous sign language recognition.

The main drawback of conventional CNN-based architectures is that they holistically involve the scene. This might not be a problem in applications such as action recognition, where most of the patterns in the scene are relevant. Unfortunately, this is not the case in sign language recognition, as most of the patterns in the scene are non-relevant. 

A few hand gesture recognition-based works overcame the challenge of non-relevant patterns indirectly, but these works still have other limitations. In one of the most recent studies, a sentence-based recognition system is presented in [27]. This system utilizes arm bands with surface electromyogram and inertial measurement sensors for data collection. An attention-based encoder–decoder structure of bidirectional LSTM and multichannel CNN is utilized for sentence modeling and prediction. The input of the encoder is a whole sentence, and the output of the decoder is a prediction probability matrix for each word in a predefined lexicon. The output sentence is inferred from the probability matrices through a language model. This prototype shows encouraging performance on 60 sentences from the Chinese sign language. Unfortunately, the user needs to maintain those armbands, which is inconvenient. 

In another recent study, LSTM and multisensory 3D data of operator’s fingers are utilized in developing a human–robot interaction prototype for surgical robot control [28]. The input data are fused, processed, and transformed to an appropriate form before utilizing the LSTM for temporal modeling and classification. The prototype showed the encoding performance on ten gestures of Chinese numbers. Such systems perform well on simple gestures, which are performed within restricted volume space. Unfortunately, it is not scalable for the highly structured gestures of sign language, which depend not only on the fingers’ configuration but also on the global position and motion of the hands with respect to the signer body.

One of the most promising solutions for this challenge is graph neural networks, in which the representative patterns in the scene can be selected easily. 

For instance, Spatial–Temporal Graph Convolutional Network (ST-GCN) is the basis for many graph-based systems for sign language recognition [29]. It utilizes the Openpose framework for skeleton joints’ extraction. Then, the nodes’ features embedding, aggregation, and updating are performed by a deep network of ST-GCN layers. The number of trainable parameters in this network is more than three million. It showed encouraging performance on action recognition of the NTU rgb+d dataset [30]. The proposed algorithm in [29] was modified to accept a custom graph layout, which is appropriate for sign language graph representation [31]. This modified version of the algorithm was evaluated on a dataset containing 20 selected classes from the ASLLVD dataset. It obtained promising results compared to the state of the art. It was also tested on the entire dataset to establish a reference for future evaluation on that dataset.

The Skeleton Aware multi-stream sign language recognition framework is one of the most recent graph-based systems for sign language recognition [32,33]. These frameworks combined the ST-GCN [31] with other input channels such as RGB frames and optical flow; in a multimodality scheme, the different modalities are integrated and fused at different levels. Even though these systems achieved excellent performance on the AUTSL dataset, the main drawback of this framework is that it is slow and involves a high computation cost. There are multiple phases for data preparation within the framework, which utilizes other complex frameworks such as the whole body pose estimation. Consequently, it cannot be deployed for real-time applications.

Other recent work employed a video transformer network (VTN) for sign language recognition [34]. VTN is a modified version of the transformer that was deployed for machine translation [35]. The cross-attention decoder of the transformer was removed, while the multi-head attention layers of the encoder were utilized as a new decoder. A separate VTN was also applied on a cropped region of the hand to enhance the resolution of hand configuration (VTN-HC). Furthermore, the pose flow was also utilized to enhance motion encoding (VTN-PF).

Most of the graph-based architectures in the literature are so deep that they perform a large number of message passing operations, which involves a large number of feature aggregations and updating. This repetition for nodes’ feature aggregation leads at a certain point to the well-known phenomenon of feature over smoothing [36]. In such a situation, the graph nodes become almost similar representations, and their contributions to the overall graph classification become minimal. To overcome this challenge, we propose a shallow architecture of decoupled 3DCNN on sign language graph representation. While reducing the number of layers and as a result the computation complexity of the proposed architecture, we enhance its performance by adding a multi-head attention layer to learn the context representation of the nodes at the frame level. 

There are other numerous works on fusion-based approaches [37,38]. Single-head attention and multi-head attention have been used in applications based on physiological signals [39,40] 

## 3. Datasets

The proposed architecture is evaluated on the KSU-SSL dataset and four other benchmark datasets for sign language recognition. These datasets vary in terms of scene complexity, number of classes, number of samples per class, and the average length of videos.

### King Saud University Saudi Sign Language (KSU-SSL) Dataset

**Figure 1:**
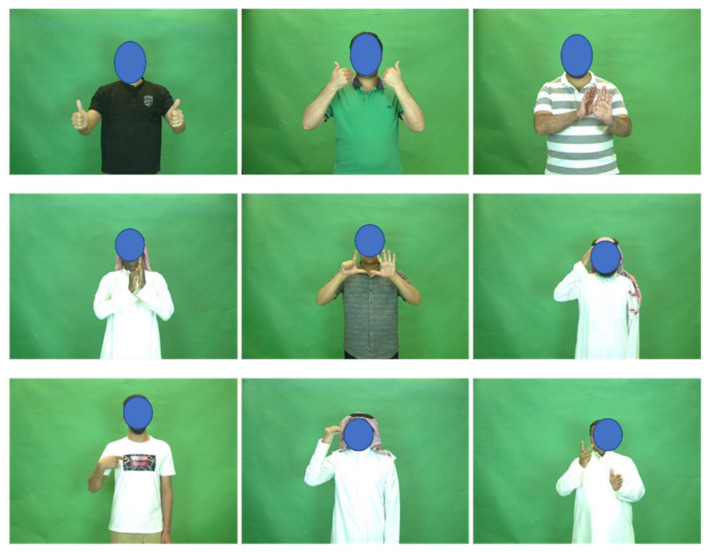
Sample frames from the KSU-SSL dataset.

The collection of the KSU-SSL dataset is still in progress. It is collected by the Center of smart robotic research at King Saud University. It consists of 293 classes from the daily life sign language gestures in the Kingdom of Saudi Arabia (KSA). The KSA sign languages have several dialects; however, we choose the one which is recommended by the Saudi Association for Hearing Impairment. It can be noted that some deaf people sometimes do not adhere to a specific dialect. In the database, we do not include such deaf people. Most of the gestures were selected from the medical field for their importance and high demand by deaf people. This dataset is recorded in a well-controlled environment with suitable lighting conditions and uniform background color. The recording studio setup utilizes three frontal imaging devices: a high-speed RGB camera, an IR camera, and a mobile camera. The recorded gestures are performed by deaf, hard-of-hearing, sign language experts, and non-deaf people. The recorded gestures are also verified by sign language experts. Each participant is invited to perform each gesture five times. The last repetition is recorded with colored hands. Until the time of conducting this study, 30 participants have completely or partially recorded the dataset gestures. In this study, only the RGB camera videos without colored hands are involved. Sample frames from the KSU-SSL dataset are illustrated in Figure 1. 

**Figure 2:**
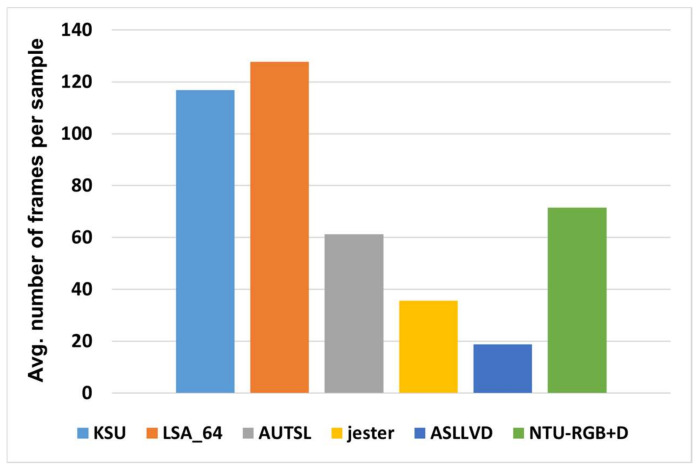
Average video length in different datasets.

The other datasets used for evaluation are the AUTSL dataset [41], the Argentinean sign language dataset (LSA64) [42], the American sign language lexicon video dataset (ASLLVD) [43], and Jester [44]. Some explanatory statistics of these datasets are summarized in Table 1. Figure 2 also illustrates how the average number of frames per video varies in different datasets.

## 4. Methodology

To build an efficient graph-based sign language recognition system, on the one hand, we proposed a lightweight 3DGCN with a low number of trainable parameters for representation learning. On the other hand, we utilized MediaPipe, which is the most efficient human landmarks estimator to extract the required graph nodes for recognition. Furthermore, other techniques such as multi-head self-attention and frame nodes’ partitioning were also utilized to boost the learning efficiency.

### 4.1. MediaPipe-Based Graph Construction

**Figure 3:**
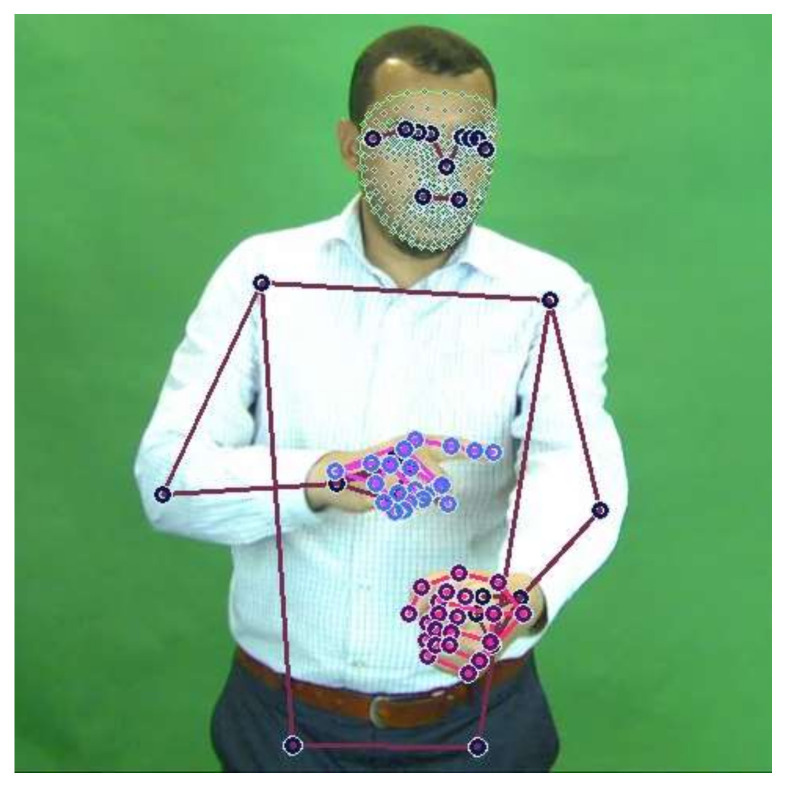
MediaPipe landmarks estimation sample.

MediaPipe is a recent framework presented by Google. It offers cross-platform, machine learning solutions for streaming media. It enables the live perception of human pose, hand tracking, and face landmarks on mobile devices and in real time [45]. Each solution enables a wide range of modern life applications such as augmented reality, fitness, and sports analysis. MediaPipe can detect and track 33 pose landmarks, 21 landmarks per hand and 468 face landmarks in the three mentioned solutions, respectively. Furthermore, MediaPipe provides a holistic solution, which integrates models for the pose, the hands, and the face components. The holistic solution can accurately estimate and track 543 landmarks in total. Each estimated landmark is represented in x, y, and z coordinates. Figure 3 depicts some of the estimated landmarks in a sample frame from the KSU-SSL dataset.

**Figure 4:**
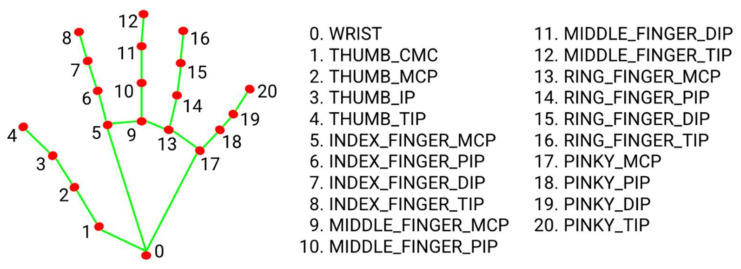
MediaPipe hand landmarks [46].

To build the sign graph, we only selected the most relevant 25 landmarks so that we can obtain an excellent recognition rate while maintaining a minimum computation complexity. From the 21 landmarks illustrated in Figure 4, for each hand, we selected ten landmarks (0, 4, 5, 8, 9, 12, 13, 16, 17, and 20). The other five selected landmarks represent the nose, the shoulders, and the elbows. The nose landmark was used as a reference to normalize the landmarks in each frame individually.

The output of this step is an undirected spatial–temporal graph $G\;=\;\left(V,\;E\right)$ with 25 nodes and T frames featuring both intra-body and inter-frame connections. The nodes set in this graph $V\;=\;\left\{v_{ti}|t\;=\;1,\ldots ,\;T,\;i\;1,\ldots ,25\right\}$ includes the selected 25 landmarks in each frame through the sequence. The nodes in one frame are connected by edges according to the connectivity of the human body structure, and each node is connected with itself in consecutive frames. Consequently, the edge set E consists of two types of edges, the intra-frame edges at each frame and the inter-frame edges, which connect the nodes in consecutive frames.

Moreover, the number of selected frames from each video sample was controlled by a predefined window size. For simplicity, the value of the window size was set based on the average number of frames per video in the dataset.

### 4.2. Graph Representation Learning

**Figure 5:**
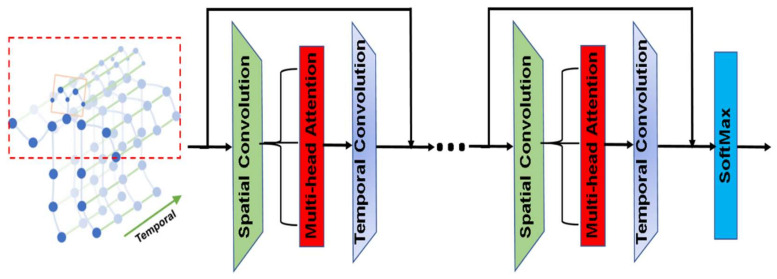
The proposed 3DGCN architecture.

The proposed architecture for representation learning consists of five consecutive layers of separable 3DGCN. As illustrated in Figure 5, the spatial and temporal convolution operations were separated by a spatial multi-head self-attention layer. The input of the layer was also passed to the output through a residual connection. For the spatial convolution, this work assumes that the spatial neighborhood of any node consists of all the nodes within a single step distance from that node.

#### 4.2.1. Basic Separable 3DGCN Implementation

The implementation of the convolution operation on graph data is not straightforward, as it is implemented on image data, which is in the form of a regular grid. Similar to most of the work in the literature, we investigate an implementation such as the one presented in [47]. Within a single frame, the nodes’ connections are represented by an adjacency matrix *A*. The self-connections of nodes are also represented by an identity matrix *I*; hence, the spatial convolution was implemented as in Equation (1).

(1) $$f_{out}\;=\;\Lambda^{-\frac{1}{2}}\left(A\;+\;I\right)\Lambda^{-\frac{1}{2}}f_{in}W$$

where $f_{out}$ is the output embedding of nodes, $\Lambda^{ii}\;=\;\sum_{j}\left(A^{ij}\;+\;I^{ij}\right)$, and the shared weight matrix for node-wise feature transformation W is formed by stacking the weight vectors of multiple kernels. The input nodes’ embedding $f_{in}$ is also represented by a tensor of the form $\left(C,\;V,\;T\right)$, where C is the number of channels, V is the number of nodes per frame, and T is the number of frames. The convolution operation in Equation (1) performs a standard 2D convolution on each frame separately and then multiplies the resulted tensors with the normalized adjacency matrix $\Lambda^{-\frac{1}{2}}\left(A\;+\;I\right)\Lambda^{-\frac{1}{2}}$. Another 2D convolution was then performed along the temporal dimension of the output tensors to model the dependency between the consecutive frames. The output layer utilized the categorical cross-entropy function for loss optimization.

#### 4.2.2. Enhanced Separable 3DGCN Implementation

A multi-head self-attention layer was added after each spatial convolution operation to enhance the nodes’ context representation in each frame. To achieve that, the adjacency matrix was scaled by a matrix of normalized attention scores of the same size. Each element in the attention matrix encoded a pair-wise normalized attention score between two neighbors. Accordingly, the new spatial embedding $h^{\left(l+1\right)}$ of the nodes was computed as follows:

1. The node features were transformed through a 2D spatial convolution as in Equation (2).
  (2) $$z^{\left(l\right)}\;=\;W^{\left(l\right)}h^{\left(l\right)}$$
  where $W^{\left(l\right)}$ is the convolution kernel and $h^{\left(l\right)}$ is the input embedding of nodes.
2. Unnormalized attention scores were computed between each pair of neighboring nodes as in Equation (3).
  (3) $$e_{ij}^{\left(l\right)}\;=\;LeakyReLU({\vec{a}}^{{\left(l\right)}^{T}}\left(z_{i\_batch}^{\left(l\right)}||z_{j\_batch}^{\left(l\right)}\right))\;$$
  where $e_{ij}^{\left(l\right)}$ is the unnormalized attention score between nodes *i* and *j*, ${\vec{a}}^{\left(l\right)}$ is a learnable weight vector, and $z_{i\_batch}^{\left(l\right)}$ and $z_{j\_batch}^{\left(l\right)}$ are the feature means of nodes *i* and *j* on the entire batch.
3. The attention scores were then normalized via SoftMax as in Equation (4). The normalized attention matrix was then used to scale the adjacency matrix.

(4) $$\alpha_{ij}^{\left(l\right)}\;=\;\frac{\exp(e_{ij}^{\left(l\right)})}{\sum_{k\in N\left(i\right)}\exp(e_{ik}^{\left(l\right)})}$$

4. Finally, the output of the convolution was multiplied by the scaled adjacency matrix to form the new embedding of the nodes as in Equation (5).

(5) $$h^{\left(l+1\right)}\;=\;\Lambda^{-\frac{1}{2}}\left(A+I\right)\Lambda^{-\frac{1}{2}}z^{\left(l\right)}$$

As in the basic approach, the temporal dependence between consecutive frames was modeled by another 2D convolution operation along the temporal dimension of the output tensors. The output layer utilized the categorical cross-entropy function for loss optimization.

Furthermore, we investigated three partitioning strategies suggested by [29] for more enhancement of spatial representation.

- The uni-labeling partitioning: All the neighboring nodes of a corresponding root node are considered as a single set, including the root node itself. The representations of all nodes are transformed by a single learnable kernel.
- The distance partitioning: The nodes are partitioned into two subsets based on their distance from the root node. The first subset includes the root node with a distance d = 0, and the second subset includes the remaining nodes with d = 1. Two different learnable kernels are utilized to transform the nodes’ representation in the two subsets.
- The spatial partitioning: The neighboring nodes are partitioned into three subsets based on their distance from the root node and the gravity center of the whole skeleton as follows: (1) the root node itself; (2) the centripetal: the set of nodes that are closer to the gravity center of the skeleton than the root node; and (3) the centrifugal: the set of nodes that are closer to the root node than the gravity center of the skeleton. In this work, the nose point was set as a reference point instead of the center of gravity.

## 5. Experimental Results and Discussion

The proposed architecture in this work was implemented using Pytorch and the training was conducted on NVIDIA RTX 3090 24 GB GPU. The recognition accuracy, which is the percentage of recognition rate, is used as an evaluation metric in our experiments. It is defined as:

(6) $$Acc\;=\;\frac{The\;number\;of\;correctly\;recognized\;samples}{Total\;number\;of\;samples\;used\;for\;evaluation}\times 100\;\%$$

### 5.1. Evaluation of the Basic 3DGCN-Based Architecture

To demonstrate the performance of the lightweight basic 3DGCN architecture, the evaluation was conducted on the KSU-SSL dataset and the other four datasets. Most of these datasets are publicly available with training and validation splits but not the test split. Hence, the reported results in this part are in terms of recognition accuracy on the validation data. The number of samples in both training and validation splits used in this work is summarized in Table 1. ASLLVD-20 is a partial dataset of 20 classes from the entire dataset ASLLVD, which contains 2745 classes. In this partitioning, we followed the same criteria in [31] to create a more balanced dataset with a small number of classes.

A fixed configuration was used to conduct the training, where mini-batch gradient descent was utilized with a batch size of 32 samples and an adapted learning rate. The initial value of the learning rate was set to 0.1 with an updating step size of 40 epochs. The learning rate was decayed after each step size according to Equation (7), which enables a smoother fine tuning for the trainable parameters with the advancement in training time.

(7) $$Lr_{new}\;=\;Lr_{current}\times \gamma$$

where γ was set to 0.5. 

**Figure 6:**
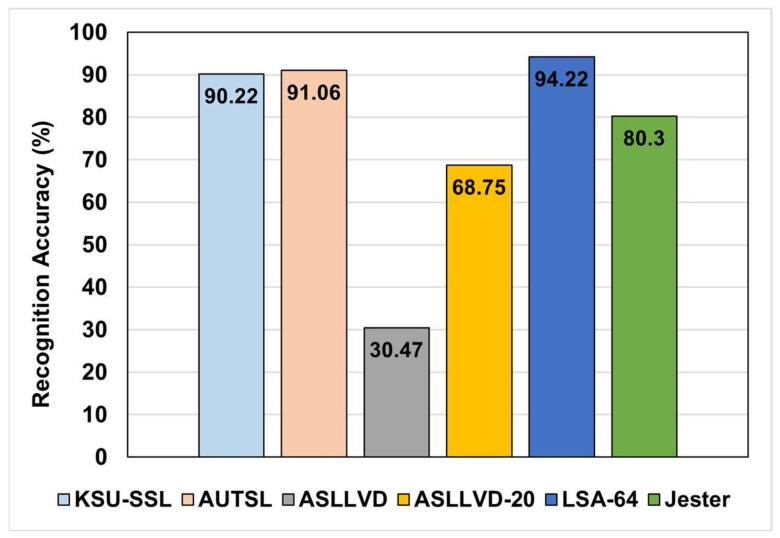
Basic architecture accuracy on different datasets.

In each experiment, the architecture was trained for 200 epochs. After each epoch, if the architecture achieved better performance, the parameter values were saved so that the final model is the one that achieved the best performance regardless of the number of training epochs. The performance of the architecture on different datasets is illustrated in Figure 6. 

Even though the basic architecture is very light and has a small number of trainable parameters, Figure 6 illustrates that the architecture was generalized well and achieved encouraging performance on most of the datasets. The highest number of trainable parameters was ≈0.3 M (in the case of the KSU-ArSL dataset). Furthermore, the architecture was able to achieve such performance despite it being trained from scratch on the original datasets without any augmentation. This encouraging performance demonstrates that graph neural networks might perform better when protected from over smoothing via reducing the repetitions of messages passing.

### 5.2. Evaluation of the Enhanced 3DGCN-Based Architecture

This experiment was started by optimizing the most important hyperparameters of the partitioning strategy and the number of self-attention heads on the KSU-SSL dataset. We conducted a grid search to optimize these two hyperparameters. The search space was defined as follows:

- The partitioning strategy $PS\in \left\{unilabling,distance,\;spatial\right\}$, where these strategies are defined in Section 4.2.2.
- The number of self-attention heads $h\in \left\{1,2,3,4\right\}$.

The result of this grid search step in terms of recognition accuracy (%) is illustrated in Table 2. 

**Figure 7:**
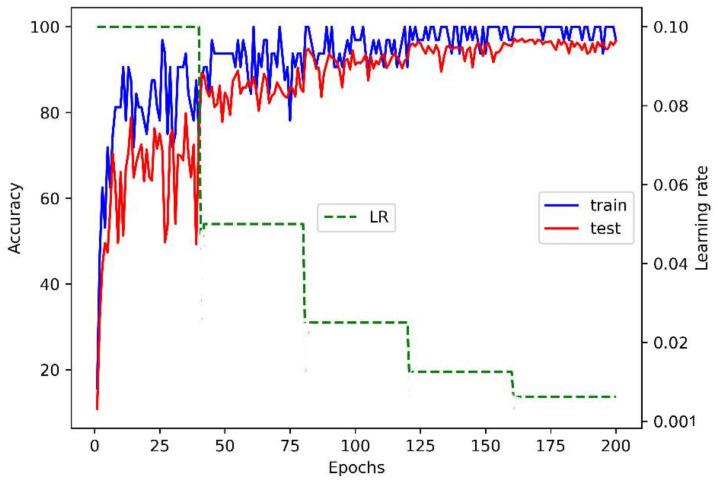
The behavior of the optimized architecture on the KSU-SSL dataset.

Figure 7 illustrates the convergence of the architecture with the optimal hyperparameters over training time on the KSU-SSL dataset. It also demonstrates how the architecture is smoothly tuned on the dataset with the regular decay in the learning rate.

**Figure 8:**
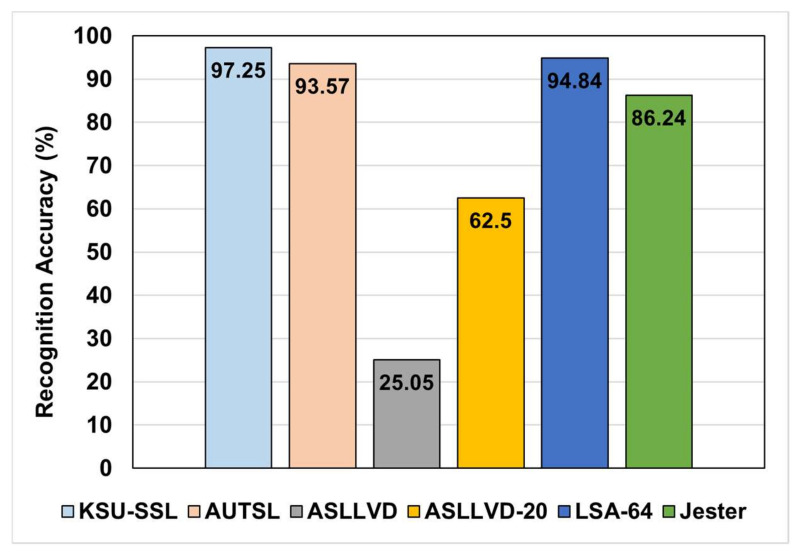
Enhanced architecture accuracy on different datasets.

After that, the optimized architecture was evaluated on the other datasets. The results are illustrated in Figure 8. It is clear from these results that the performance of the proposed architecture was significantly enhanced by the multi-head attention layer on the KSU-SSL, AUTSL, and Jester datasets. These three datasets are comprehensive in terms of the number of classes and the number of samples in each training and validation split, as shown in Table 1. The comprehensiveness of these datasets benefited from the increased number of parameters in the multi-head attention layer. The multi-head attention parameters generalized the architecture and enabled it to reflect the large variety in sign configuration at the frame level. 

Furthermore, it is noticed that the performance enhancement of the architecture on the LSA-64 is very slight, which might be attributed to the fact that LSA-64 was recorded under very restricted conditions regarding the background homogeneity, and there was light consistency in addition to using colored gloves. These restricted conditions enabled even conventional CNN models to achieve high accuracies on this dataset, as the relevant patterns are easily distinguished from the non-relevant background. In such a situation, the contribution of the spatial attention layer is minimal. On the other hand, the performance of the enhanced architecture was worse on both versions of the ASLLVD dataset. This bad performance can be attributed to the size of the training data. As shown in Table 1, there are a limited number of samples for training relative to the number of classes in the dataset, which lead both the basic and enhanced architectures to overfitting. The situation became worse with the increased number of learnable parameters in the enhanced architecture, which makes the generalization more difficult.

The proposed architecture was compared with the state-of-the-art graph-based architecture on both AUTSL and ASLLVD datasets. In Table 3, the performance of the proposed architecture is compared with the reported results for different variants of the VTN architecture on the AUTSL dataset [34]. From Table 3, we can observe that the proposed architecture with spatial attention enhancement outperformed the best variant of VTN (VTN-PF) on both the validation and test datasets. Moreover, the number of trainable parameters in the proposed architecture is nearly one-hundredth the number of parameters of VTN architecture.

Similarly, Table 4 shows that the proposed architecture outperformed the ST-GCN architecture [31], with large margins on both the entire ASLLVD dataset and the selected 20 classes dataset. Even though the performance of the architecture was degraded by adding the attention layer, it still outperformed the ST-GCN architecture on both datasets. The performance degradation after the addition of the attention layer is expected because of the increased effect of overfitting, since we nearly doubled the number of trainable parameters while keeping training on the same number of training samples.

Even though the proposed architecture achieved encouraging performance, it still has some limitations. The most important limitation of this architecture is the presumption that only the sign performer should be in the scene. The performance degrades dramatically when multiple persons appear in the scene. This limitation is common in all human skeletal-based systems. For that reason, the performance of such systems in action recognition is very limited on the Kinetics dataset as in [29]. The assumption of presenting a single performer in the scene is reasonable in the case of sign language recognition. Unfortunately, it is inapplicable in the case of action recognition, as many actions involve an interaction between multiple persons.

## 6. Conclusions

A lightweight 3DGCN architecture is proposed in this study for sign language recognition. The proposed architecture utilizes a few 3DGCN layers to avoid the common over-smoothing effect in deep GCN architectures, which results from the high repetitions of messages passing between the graph nodes. This shallow architecture is utilized to construct a graph representation from the most relevant MediaPipe landmarks of the signer body. This embedding step transfers the recognition problem to graph classification. Reducing the depth of the architecture might intuitively lead to less efficient representation, but we substitute that by enhancing the spatial representation with less computation cost. To achieve that, a spatial attention mechanism is added to the proposed architecture to enhance the modeling of the spatial patterns of gestures. The 3DGCN layers are decoupled into a spatial and temporal convolution, which are separated by a spatial multi-head self-attention layer. This added layer enhances the local representation in each frame rather than modeling the global dependence of the frames. The proposed architecture is evaluated on various datasets of different characteristics and compared to state-of-the-art architectures. Our experiments demonstrate the effectiveness of the spatial attention layer to enhance the performance of such a lightweight 3DGCN architecture. Moreover, the proposed architecture shows outstanding performance and generalizes well on various datasets. 

For future work, the attention mechanism will be utilized in different ways to enhance the temporal molding of the gestures toward a greater generalization of the architecture. The proposed architecture will also be prototyped and evaluated in a real-time sign language recognition scenario.

## Figures and Tables

**Table 1:** Statistics of different datasets used in this study.

Dataset	Num. of Classes	Num. of Training Samples	Num. of Validation Samples
KSU-SSL	293	28,021	5860
AUTSL	226	28,142	4418
ASLLVD-20	20	85	42
ASLLVD	2745	7798	1950
SLA-64	64	2560	640
Jester	27	118,558	14,786

**Table 2:** Results (% accuracies) of the hyperparameters’ optimization.

		Partitioning		
		*Uni-Labeling*	*Distance*	*Spatial*
No. of heads	1	96.62	96.77	96.79
	2	96.7	96.82	96.52
	3	97.08	96.93	96.86
	4	96.57	97.03	**97.25**

**Table 3:** Performance comparison on the AUTSL dataset.

Architecture	Validation Acc. (%)	Test Acc. (%)	Num. of Params (×10^6^)
VTN	82.03	-	≈29
VTN-HC	90.13	-	≈51
VTN-PF	91.51	92.92	≈52
Basic 3DGCN (ours)	91.06	90.27	≈0.3
Enhanced 3DGCN (ours)	93.57	**93.38**	≈0.7

**Table 4:** Performance comparison on the ASLLVD dataset.

Architecture	Dataset	Validation Acc. (%)
ST-GCN	ASLLVD-20	61.04
Basic 3DGCN (ours)		**68.75**
Enhanced 3DGCN (ours)		**62.5**
ST-GCN	ASLLVD	16.48
Basic 3DGCN (ours)		**30.47**
Enhanced 3DGCN (ours)		**25.05**

## References

[1] Agrawal S., Jalal A., Tripathi R. (2016). A survey on manual and non-manual sign language recognition for isolated and continuous sign. Int. J. Appl. Pattern Recognit..

[2] Rautaray S., Agrawal A. (2015). Vision based hand gesture recognition for human computer interaction: A survey. Artif. Intell. Rev..

[3] Zhang X., Chen X., Li Y., Lantz V., Wang K., Yang J. (2011). A framework for hand gesture recognition based on accelerometer and EMG sensors. IEEE Trans. Syst. Man Cybern. A Syst. Hum..

[4] Côté-Allard U., Fall C.L., Drouin A., Campeau-Lecours A., Gosselin G., Glette K., Gosselin B. (2019). Deep learning for electromyographic hand gesture signal classification using transfer learning. IEEE Trans. Neural Syst. Rehabil. Eng..

[5] Al-Hammadi M., Muhammad G., Abdul W., Alsulaiman M., Bencherif M., Mekhticheand M.A. (2020). Hand gesture recognition for sign language using 3DCNN. IEEE Access.

[6] Altaheri H., Muhammad G., Alsulaiman M., Amin S.U., Altuwaijri G.A., Abdul W., Bencherif M.A., Faisal M. (2021). Deep learning techniques for classification of electroencephalogram (EEG) motor imagery (MI) signals: A review. Neural Comput. Appl..

[7] Hossain M., Al-Hammadi M., Muhammad G. (2018). Automatic fruit classification using deep learning for industrial applications. IEEE Trans. Ind. Inform..

[8] Su H., Qi W., Hu Y., Karimi H.R., Ferrigno G., Momi E.D. (2020). An incremental learning framework for human-like redundancy optimization of anthropomorphic manipulators. IEEE Trans. Ind. Inform..

[9] Qi W., Su H. (2022). A cybertwin based multimodal network for ecg patterns monitoring using deep learning. IEEE Trans. Ind. Inform..

[10] Cao Z., Simon T., Wei S.E., Sheikh Y. OpenPose: Realtime Multi-Person 2D Pose Estimation using Part Affinity Fields. Proceedings of the 2017 IEEE Conference on Computer Vision and Pattern Recognition.

[11] Selvaraj P., NC G., Kumar P., Khapra M. (2021). OpenHands: Making Sign Language Recognition Accessible with Pose-based Pretrained Models across Languages. arXiv.

[12] Yasir F., Prasad P.W.C., Alsadoon A., Elchouemi A. Sift based approach on bangla sign language recognition. Proceedings of the IEEE 8th International Workshop on Computational Intelligence and Applications (IWCIA).

[13] Thrwat A., Gaber T., Hassanien A.E., Shahin M.K., Refaat B. (2015). Sift-based arabic sign language recognition system. Adv. Intell. Syst. Comput..

[14] Liwicki S., Everingham M. Automatic recognition of fingerspelled words in british sign language. Proceedings of the IEEE Computer Society Conference on Computer Vision and Pattern Recognition Workshops.

[15] Buehler P., Zisserman A., Everingham M. Learning sign language by watching tv (using weakly aligned subtitles). Proceedings of the IEEE Conference on Computer Vision and Pattern recognition.

[16] Badhe P., Kulkarni V. Indian sign language translator using gesture recognition algorithm. Proceedings of the IEEE International Conference on Computer Graphics.

[17] Starner T., Weaver J., Pentland A. (1998). Real-time american sign language recognition using desk and wearable computer based video. IEEE Trans. Pattern Anal. Mach. Intell..

[18] Lichtenauer J.F., Hendriks E.A., Reinders M.J. (2008). Sign language recognition by combining statistical DTW and independent classification. IEEE Trans. Pattern Anal. Mach. Intell..

[19] Nagarajan S., Subashini T.S. (2013). Static hand gesture recognition for sign language alphabets using edge oriented histogram and multi class SVM. Int. J. Comput..

[20] Tornay S., Razavi M., Doss M. Towards multilingual sign language recognition. Proceedings of the IEEE International Conference on Acoustics, Speech and Signal Processing (ICASSP).

[21] Pigou L., Oord A., Dieleman S., Herreweghe M.V., Dambre J. (2018). Beyond temporal pooling: Recurrence and temporal convolutions for gesture recognition in video. Int. J. Comput. Vis..

[22] Chen X., Gao K. (2018). DenseImage network: Video spatial-temporal evolution encoding and understanding. arXiv.

[23] Al-Hammadi M., Muhammad G., Abdul W., Alsulaman M., Hossain M.S. (2019). Hand gesture recognition using 3D-CNN model. IEEE Consum. Electron. Mag..

[24] Liu Y., Jiang D., Duan H., Sun Y., Li G., Tao B., Yun J., Liu Y., Chen B. (2021). Dynamic gesture recognition algorithm based on 3D convolutional neural network. Comput. Intell. Neurosci..

[25] Al-Hammadi M., Muhammad G., Abdul W., Alsulaman M., Bencherif M.A., Alrayes T.S., Mekhtiche M.A. (2020). Deep learning-based approach for sign language gesture recognition with efficient hand gesture representation. IEEE Access.

[26] Qin W., Mei X., Chen Y., Zhang Q., Yao Y., Hu S. Sign Language Recognition and Translation Method based on VTN. Proceedings of the International Conference on Digital Society and Intelligent Systems.

[27] Wang Z., Zhao T., Ma J., Chen H., Liu K., Shao H., Wang Q., Ren J. (2022). Hear sign language: A real-time end-to-end sign language recognition system. IEEE Trans. Mob. Comput..

[28] Qi W., Ovur S.E., Li Z., Marzullo A., Song R. (2021). Multi-Sensor Guided Hand Gesture Recognition for a Teleoperated Robot Using a Recurrent Neural Network. IEEE Robot. Autom. Lett..

[29] Yan S., Xiong Y., Lin D. Spatial temporal graph convolutional networks for skeleton-based action recognition. Proceedings of the Thirty-Second AAAI Conference on Artificial Intelligence.

[30] Shahroudy A., Liu J., Ng T., Wang G. Ntu rgb+d: A large scale dataset for 3d human activity analysis. Proceedings of the IEEE Conference on Computer Vision and Pattern Recognition.

[31] Amorim C.C.D., Macêdo D., Zanchettin C. Spatial-temporal graph convolutional networks for sign language recognition. Proceedings of the International Conference on Artificial Neural Networks.

[32] Jiang S., Sun J., Wang L., Bai Y., Li K., Fu Y. Skeleton aware multi-modal sign language recognition. Proceedings of the IEEE/CVF Conference on Computer Vision and Pattern Recognition.

[33] Jiang S., Sun B., Wang L., Bai Y., Li K., Fu Y. (2021). Sign Language Recognition via Skeleton-Aware Multi-Model Ensemble. arXiv.

[34] Coster M.D., Herreweghe M.V., Dambre J. Isolated sign recognition from rgb video using pose flow and self-attention. Proceedings of the IEEE/CVF Conference on Computer Vision and Pattern Recognition.

[35] Vaswani A., Shazeer N., Parmar N., Uszkoreit J., Jones L., Gomez A.N., Kaiser L., Polosukhin L. Attention is all you need. Proceedings of the Advances in Neural Information Processing Systems.

[36] Zhou K., Huang X., Li Y., Zha D., Chen R., Hu X. (2020). Towards deeper graph neural networks with differentiable group normalization. arXiv.

[37] Muhammad G., Alshehri F., Karray F., Saddik A.E., Alsulaiman M., Falk T. (2021). A comprehensive survey on multimodal medical signals fusion for smart healthcare systems. Inf. Fusion.

[38] Muhammad G., Hossain M. (2021). COVID-19 and non-COVID-19 classification using multi-layers fusion from lung ultrasound images. Inf. Fusion.

[39] Altuwaijri G., Muhammad G., Altaheri H., Alsulaiman M. (2022). A Multi-Branch Convolutional Neural Network with Squeeze-and-Excitation Attention Blocks for EEG-Based Motor Imagery Signals Classification. Diagnostics.

[40] Amin S., Altaheri H., Muhammad G., Abdul W., Alsulaiman M. (2022). Attention-Inception and Long Short-Term Memory-based Electroencephalography Classification for Motor Imagery Tasks in Rehabilitation. IEEE Trans. Ind. Inform..

[41] Sincan O.M., Keles H.Y. (2020). Autsl: A large scale multi-modal turkish sign language dataset and baseline methods. IEEE Access.

[42] Ronchetti F., Quiroga F., Estrebou C., Lanzarini L., Rosete A. LSA64: An Argentinian sign language dataset. Proceedings of the XXII Congreso Argentino de Ciencias de la Computación.

[43] Neidle C., Thangali A., Sclaroff S. Challenges in Development of the American Sign Language Lexicon Video Dataset (ASLLVD) Corpus. Proceedings of the Conference Language Resources and Evaluation Conference (LREC).

[44] Materzynska J., Berger G., Bax I., Memisevic R. The jester dataset: A large-scale video dataset of human gestures. Proceedings of the IEEE/CVF International Conference on Computer Vision Workshop (ICCVW).

[45] Lugaresi C., Tang J., Nash H., McClanahan C., Uboweja E., Hays M., Zhang F., Chang C., Yong M., Lee J. MediaPipe: A Framework for Perceiving and Processing Reality. Proceedings of the Third Workshop on Computer Vision for AR/VR at IEEE Computer Vision and Pattern Recognition.

[46] Google Research Team (2020). MediaPipe. https://google.github.io/mediapipe/solutions/hands.html.

[47] Kipf T., Welling M. (2016). Semi-supervised classification with graph convolutional networks. arXiv.

